# Supplementary material for: Drops of Capillary Blood Are Not Appropriate for Hemoglobin Measurement with Point-of-Care Devices: A Comparative Study Using Drop Capillary, Pooled Capillary, and Venous Blood Samples
Source: Nutrients. 2022 Dec 16;14(24):5346. doi: 10.3390/nu14245346 (PMC9786209; doi:10.3390/nu14245346)
Supplement: Supplementary file 1 [file nutrients-14-05346-s001.zip › nutrients-2079606-supplementary.pdf]

Supplementary Material

Table of Contents

Table S1. Bias from Bland-Altman and linear correlation parameters of venous blood hemoglobin measurements using each HemoCue apparatus *versus* the cyanmethemoglobin method 2

Figure S1. Bland Altman plot and box plot of hemoglobin (Hb) measurement difference (g/dL) in capillary *versus* venous blood samples as analyzed using the cyanmethemoglobin method. 3

Table S2. Bland-Altman plots of hemoglobin concentration determined using the HemoCue in different blood sample sources versus using the cyanmethemoglobin method with venous blood by age group. 4

Figure S2. Concordance plots by age group and sampling method 5

Figure S3. Frequency distributions of the difference between adjusted* hemoglobin measurement results using the HemoCue in three types of blood sample sources, against the cyanmethemeglobin method in venous blood 6

Table S3. Mixed linear regression model* for differences in hemoglobin (Hb) sampling methods 7

Table S4. Bland Alman of the Hb mean differences to venous blood in the reference method*, by number of measurement using HemoCue 9

Table S5. Random effects of the mixed linear regression model on differences in hemoglobin measurement 9

Table S1. Bias from Bland-Altman and linear correlation parameters of venous blood hemoglobin measurements using each HemoCue apparatus *versus* the cyanmethemoglobin method

Number of HemoCue apparatus*	Bland-Altman bias	Intercept	Slope	n
**1**	0.345	.0025454	1.027402	80
**2**	0.365	0.8078831	0.9651042	34
**3**	0.332	0.0229337	1.023791	68
**4**	0.423	−0.3411198	1.06057	26
**5**	0.400	−2.380941	1.227373	26
**6**	0.148	−0.1218974	1.021328	64
**All**	0.314	0.024098	1.022877	298

**Each HemoCue was used for only one person; these results represent the combined effect of apparatus and personnel skills.*

Figure S1. Bland Altman plot and box plot of hemoglobin (Hb) measurement difference (g/dL) in capillary *versus* venous blood samples as analyzed using the cyanmethemoglobin method.

A) Bland Altman Plot	B) Box plot
Mean ∂ Hb= 0.097 ± 0.426, 95%CI (−0.737,0.931) 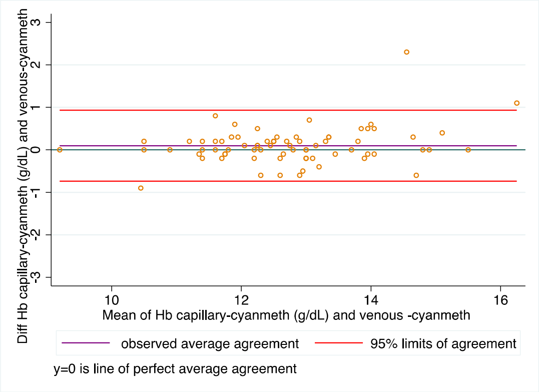	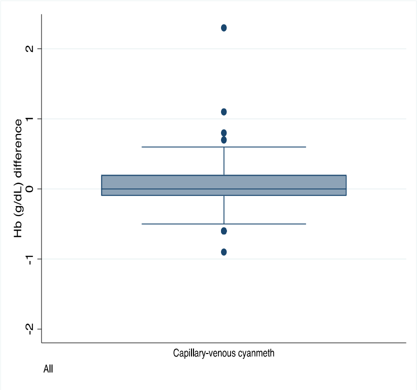

Table S2. Bland-Altman plots of hemoglobin concentration determined using the HemoCue in different blood sample sources versus using the cyanmethemoglobin method with venous blood by age group**.**

Age group	Sampling method using HemoCue	Concordance	Pearson correlation	Relative bias	Hb mean difference (g/dL)	SD
Children	Venous	0.943	0.948	0.994	−0.007	0.311
	Pool (first and second lecture)	0.867	0.87	0.997	−0.064	0.395
	Drop (second and third drop)	0.557	0.612	0.942	0.096	1.052
Women	Venous	0.978	0.98	0.998	−0.02	0.307
	Pool (first and second lecture)	0.971	0.972	0.999	−0.04	0.337
	Drop (second and third drop)	0.886	0.968	0.916	0.624	0.483
Older Adults	Venous	0.973	0.973	1.0	0.026	0.373
	Pool (first and second lecture)	0.958	0.959	0.999	0.036	0.371
	Drop (second and third drop)	0.864	0.915	0.944	0.554	0.722

Figure S2. Concordance plots by age group and sampling method

	Preschoolers	Women	Elderly
Venous	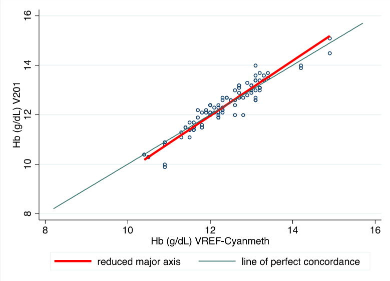	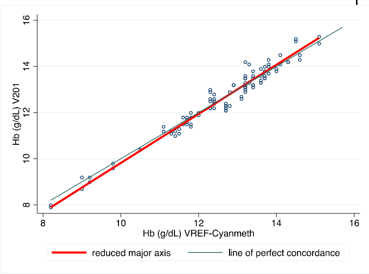	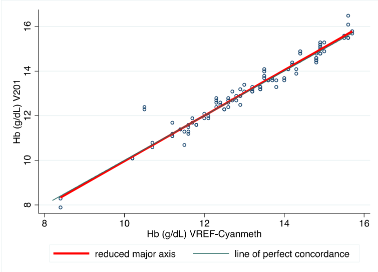
Pooled capillary blood	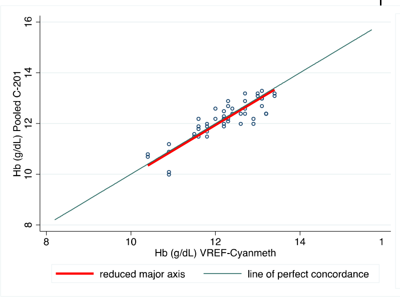	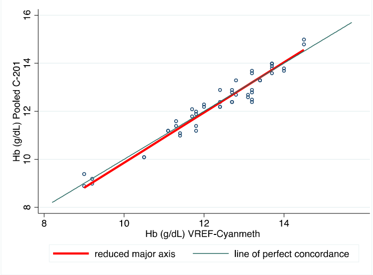	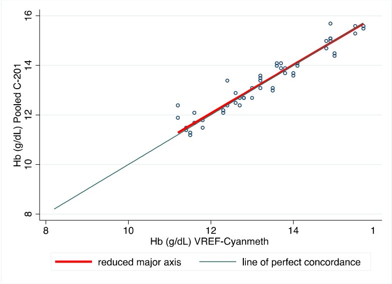
Drops of capillary blood	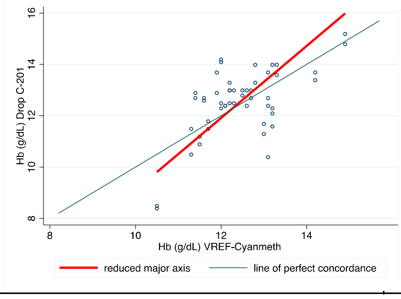	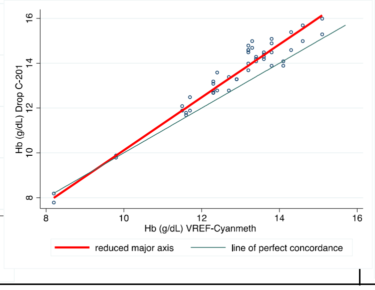	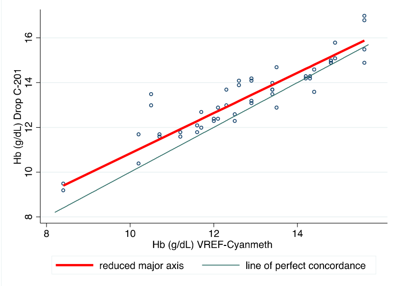

Figure S3. Frequency distributions of the difference between adjusted* hemoglobin measurement results using the HemoCue in three types of blood sample sources, against the cyanmethemeglobin method in venous blood

A) Before adjustment

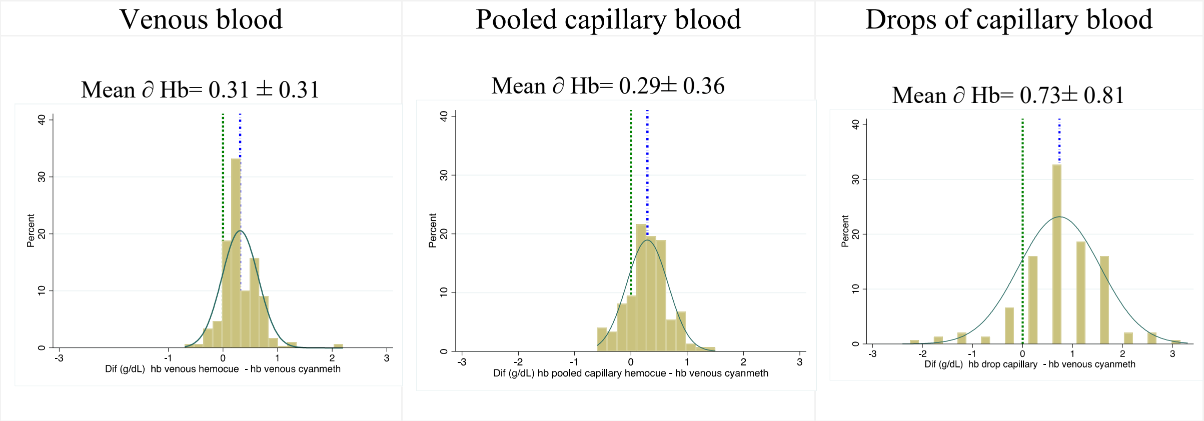

B) After adjustment

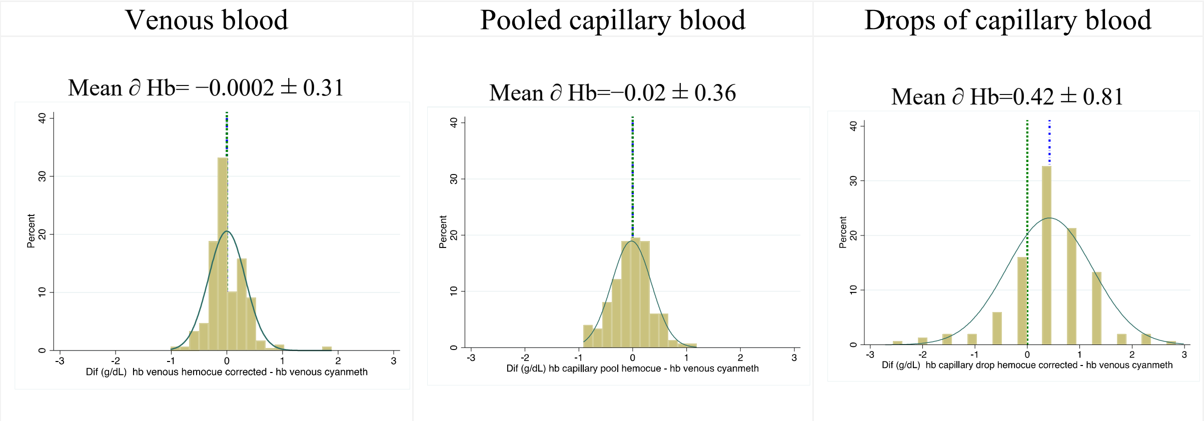

** The hemoglobin values from the HemoCue were adjusted by subtracting the average bias of 0.314 g/dL*

Table S3. Mixed linear regression model* for differences in hemoglobin (Hb) sampling methods

***Before adjustment**

	All			Children			Women			Older Adults		
	**Coef**	***p*-value**	**95%CI**	**Coef**	***p*-value**	**95%CI**	**Coef**	***p*-value**	**95%CI**	**Coef**	***p*-value**	**95%CI**
**Sampling method (ref: venous cyanmeth.)**												
Venous ABX hemocounter	0.08	0.059	(-0.003 0.15)	0.06	0.506	(-0.11, 0.22)	0.1	0.065	(-0.01, 0.21)	0.07	0.274	(-0.06, 0.2)
Capillary cyanmethemoglobin	0.09	0.09	(-0.01, 0.19)	-0.05	0.621	(-0.27, 0.16)	0.18	0.018	(0.03, 0.33)	0.14	0.096	(-0.03, 0.32)
Venous HemoCue	0.26	<0.001	(0.17, 0.35)	0.24	0.009	(0.06, 0.42)	0.22	<0.001	(0.09, 0.34)	0.3	<0.001	(0.16, 0.45)
Pooled capillary HemoCue	0.28	<0.001	(0.19, 0.37)	0.23	0.012	(0.05, 0.41)	0.26	<0.001	(0.13, 0.38)	0.35	<0.001	(0.21, 0.5)
Drop capillary HemoCue	0.74	<0.001	(0.65, 0.83)	0.43	<0.001	(0.25, 0.6)	0.95	<0.001	(0.83, 1.07)	0.86	<0.001	(0.72, 1.01)
**Intercept**	12.2	<0.001	(11.67, 12.7)	12.28	<0.001	(11.9, 12.66)	12.7	<0.001	(12.15, 12.99)	14.7	<0.001	(13.42, 15.92)

***After adjustment**

	All			Children			Women			Older Adults		
	**Coef**	***p*-value**	**95%CI**	**Coef**	***p*-value**	**95%CI**	**Coef**	***p*-value**	**95%CI**	**Coef**	***p*-value**	**95%CI**
**Sampling Method (ref: venous cyanmeth)**												
Venous ABX hemocounter	0.08	0.059	(0, 0.16)	0.06	0.506	(-0.11, 0.22)	0.10	0.065	(-0.01, 0.21)	0.07	0.274	(-0.06, 0.2)
Capillary cyanmethemoglobin	0.09	0.09	(-0.01, 0.2)	-0.05	0.621	(-0.27, 0.16)	0.18	0.018	(0.03, 0.33)	0.14	0.096	(-0.03, 0.32)
Venous HemoCue	-0.06	0.219	(-0.15, 0.03)	-0.07	0.434	(-0.25, 0.11)	-0.09	0.135	(-0.22, 0.03)	-0.01	0.923	(-0.15, 0.14)
Pooled Capillary HemoCue	-0.03	0.534	(-0.12, 0.06)	-0.08	0.383	(-0.26, 0.1)	-0.05	0.409	(-0.18, 0.07)	0.04	0.577	(-0.1, 0.18)

Drop capillary HemoCue	0.43	<0.001	(0.34, 0.52)	0.11	0.218	(-0.07, 0.29)	0.64	<0.001	(0.51, 0.76)	0.55	<0.001	(0.41, 0.69)
**Intercept**	12.2	<0.001	(11.67, 12.7)	12.28	<0.001	(11.9, 12.66)	12.58	<0.001	(12.15, 13)	14.7	<0.001	(13.42, 15.92)

**Adjusted by age and sex*

Table S4. Bland Alman of the Hb mean differences to venous blood in the reference method*, by number of measurement using HemoCue

Number of Hb measurement in HemoCue	Venous<br>∂ ± SD, (95%CI)	Pooled capillary<br>∂ ± SD, (95%CI)	Drop capillary<br>∂ ± SD, (95%CI)
First	-0.044±0.30, (-0.636, 0.549)	-0.034± 0.33, (-0.687, 0.619)	0.350±0.81, (-1.128, 1.98)
Second	0.043±0.35, (-0.650, 0.736)	-0.010± 0.40, (-0.798, 0.778)	0.49±0.82, (-1.121, 2.120)
Mean	0.000±0.30, (-0.599, 0.598)	-0.034±0.33, (-0.687, 0.619)	0.35±0.80, (-1.228, 1.928)

* *Venous blood samples analyzed using the cyanmethemoglobin method.*

Table S5. Random effects of the mixed linear regression model on differences in hemoglobin measurement

	**σ²**		**% of Total variance**	
**Group of population**	**Subject**	**Residual**	**Subject**	**Residual**
All	1.67	0.12	93.3%	6.7%
Children	0.79	0.16	82.5%	17.5%
Women	2.25	0.07	97.0%	3.0%
Older adults	1.95	0.10	92.7%	7.3%
